# PPI in research: a reflection from early stage researchers

**DOI:** 10.1186/s40900-019-0170-2

**Published:** 2019-11-19

**Authors:** Alice M. Biggane, Maria Olsen, Paula R. Williamson

**Affiliations:** 1 Department of Biostatistics, University of Liverpool, Liverpool, UK; 2 Université de Paris, CRESS, INSERM, INRA, F-75004 Paris, France; 3 Department of Clinical Epidemiology, Biostatistics and Bioinformatics, Amsterdam Public Health Research Institute, Amsterdam University Medical Centers, Meibergdreef 9, 1105 AZ Amsterdam, The Netherlands

**Keywords:** Patient and public involvement (PPI), Early stage researchers, Research on research, Waste in research, Education, Funding

## Abstract

**Background:**

The importance of patient and public involvement (PPI) in the design and conduct of health research projects is gaining widespread recognition; however, it is still a developing area. Furthermore, PPI in methodological health research can help increase research value Thus, it is of great importance that researchers, especially early stage researchers continue to discuss and learn about the future challenges and opportunities of PPI.

**Objective:**

With this commentary, we aim to disseminate i) key messages from a recent PPI training event and ii) discuss what early stage researchers (ESRs) in the “Methods in Research on Research” (MiRoR) project can do to improve our current and future work by considering and incorporating PPI.

**Main body:**

The latest MiRoR network meeting held at the University of Split in Croatia (2nd-3rd October), included a PPI training session with presentations from Mr. Stephens a patient, about “*Waste in research*” and Dr. Westmore a funder on “*Research integrity*”, followed by smaller round-table discussions. This provided early stage researchers (ESRs) with an opportunity to discuss and explore the benefits and challenges of PPI in research, and the appropriate questions and research that is required for improving the implementation of PPI in clinical research.

**Conclusion:**

As with intervention research, PPI is also important for methodological research since this will help to increase both the value, integrity and quality of research.

By providing early stage researchers with appropriate educational, interactive and real-world training, this will introduce the various merits and challenges associated with PPI in early-stage research.

## Plain English summary

Patient and public involvement (PPI) in clinical research has achieved much recognition, and there is ongoing exploration and development in the field. In this context, education and training opportunities are of paramount importance for the research community to achieve collaborations that are more fruitful. In particular for Early Stage Researchers (ESRs), as they are researchers still in training and thus, represent the next generation of scientific research.

The Methods in Research on Research (MiRoR) project is a consortium brought together by their interest in tackling waste in clinical research, by exploring topics for methodological improvement across a range of clinical research areas.

Drawing on a recent PPI training event held by the MiRoR project, we describe the learning outputs that we, the 15 MiRoR ESRs attending the event took home with us. The outputs enabled us as ESRs to learn more about the value of PPI and when and how public contributors can be involved in methodological research.

At this training event two leading experts in PPI (Mr Stephens, from The National Cancer Research Institute Consumer Forum) and funding (Dr Westmore, from the National Institute for Health Research) shared their knowledge and experiences of waste in research from a patient perspective and on research integrity from a funder’s perspective, respectively, via presentations, training and round-table discussions. This led to further consideration of what the MiRoR consortium can do as a group to enhance our partnerships with patient and public contributors and share our methodological research with the public.

## Background

In 2009, Chalmers and Glasziou estimated that 85% of all clinical research is wasted despite large financial investments, including public funding [1]. They identified four stages within current research practises that lead to waste in research including: i) prioritising research questions that are irrelevant to health professionals and patients ii) conducting unnecessary or inappropriate studies or study designs, iii) failing to publish research findings and iv) selective reporting of research findings [1].

Ultimately, all this waste in clinical research has a detrimental impact on patients and members of the public: it prevents clinicians from using effective health interventions in practice and researchers cannot adequately prioritise future research questions.

Moreover, research waste is linked with ‘research integrity’, which is defined by the National Institutes of Health (NIH) as “the use of honest and verifiable methods in proposing, performing, and evaluating research; reporting research results with particular attention to adherence to rules, regulations, guidelines, and following commonly accepted professional codes or norms” [2]. Thus, when inappropriate research priorities and practices are used, this calls into question the integrity of the research. Thus, when researchers, fail to ensure research integrity, this raises the question of whether the research is unethical. However, addressing ethics is beyond the scope of this commentary and we refer to discussion elsewhere [3, 4].

In 2014 The Lancet journal published a series of papers surrounding five sources of avoidable waste in research. In this series to authors advocated for greater consideration of research priorities [5], improved research design, conduct and analysis [6] obtaining appropriate regulatory and governance approvals [7], accessible research documentation [8] and appropriate research reporting [9].

In the wake of this increasing awareness about research waste and issues in ensuring research integrity, a new discipline of ‘research on research’ (also known as methodological-, secondary- and meta-research) emerged. By investigating research waste within the five sources mentioned above in more detail, we can develop and implement better research methods. Thus, this type ‘research on research’ aims to reduce research waste and ensure/improve research integrity.

In contrast to primary research in which studies are conducted with patients and often with outcome measures that are directly linked to patients e.g. changes in disease/health status, methodological research studies concern research methods used by other studies. Moreover, methodological research often investigates outcomes not directly linked to patient priorities or needs but on surrounding areas of the research process, such as the development of tools and guidelines for scientific reporting, peer-review process, and assessment tools for various phases of research studies.

A reduction of wasteful research is possible if the evidence that the research produces is valid and relevant to its users, including patients and members of the public [5]. The importance of patient and public involvement (PPI) in the design and conduct of projects is gaining widespread recognition [10]. PPI is important for several reasons. Firstly, patients’ lived experience and knowledge adds value in shaping research. Secondly, it is moral imperative that the voice of PPI has an impact on the research that will affect them, lastly, PPI is a way of recognising that patients are active, engaged individuals, as also expressed by INVOLVE’s definition of public involvement as: “*research being carried out ‘with’ or ‘by’ members of the public rather than ‘to’, ‘about’ or ‘for’ them.*” [11]. Thus, the collaboration between patients and researchers has been described as reflecting, “*a fundamental paradigm shift in health and social care research, away from paternalism towards partnership*” [12]. After all, doctors know about the illness and research but patients know about the daily impact of living with the health condition.

We believe this illustrates how PPI can improve the quality of research. If researchers are well informed about this importance and qualities of PPI, PPI will not only help to reduce waste, but equally improve the research integrity.

Furthermore, funding bodies are also becoming increasingly aware of the importance of PPI. In recent times, many funders stipulate that researchers must demonstrate how members of the public were involved in the design and development of the grant application, and if funded, how they will be actively involved in the planning and/or conduct of the study [11, 13].

PPI can occur at multiple stages of the research cycle, from identifying the research question, funding application, design, conduct and analysis, to dissemination and translation of findings into daily health services [14–16]. To date, PPI in research has mainly focussed on ensuring patients are equal stakeholders in an expert-dominated environment and integrating their lived experience and knowledge into clinically scientific studies [17–19]. Efforts to improve PPI, such as the experience and reporting of involvement and how different types of public contributors can add value, have been made [20–22], yet it is still a developing area and with much debate about its definitions, methods, operations, research integrity and ethical standards [23].

There are numerous ways of facilitating PPI including community involvement, PPI presence on committees or management groups, patient research partners; the most appropriate of which is likely influenced by the specific research question, health condition, population, and the available resources [15]. However, despite no one-size-fits-all approach, we believe that involving public contributors, unless there is a good reason not to, should be the default position of all research teams. Thus, ensuring PPI in research is standard practice, yet not forcing a wrongful fit.

Thus, there are some common threads that all researchers should consider before and during the research process when including PPI contributors. This includes threads such as who to involve as research partners, when to involve PPI contributors, how to access and keep people involved in project, training and support mechanisms for PPI members, follow-up plans and dissemination approaches. It is important that research teams carefully consider, understand and acknowledge these issues. Failure to do so has the potential to cause unfavourable impact thus leading to more waste in research. One example of an unfavourable impact is tokenism, which is described as the “superficial and disingenuous” inclusion of small numbers of patients, with limited involvement and impact on the research [24–27]. Thus, it is becoming ever more apparent that researchers need to learn about and consider PPI as an aspect of health research throughout their career.

Early-stage researchers (ESRs) represent the next generation of scientific research. ESR’s are typically researchers in the first 4 years of their research activity, including the period of research training. Hence, it is important that they develop and foster PPI-related awareness, skills and expertise. This will enable them to champion meaningful PPI, and to produce more valid and relevant research evidence [28]. Moreover, if the skills and values of PPI are introduced earlier in their training, it will help them adopt these practices better and thus, the involvement of public contributors will become a natural way of working, rather than a ‘tick box’ exercise.

The Methods in Research on Research (MiRoR) consortium is a training programme in the field of methods in clinical research. MiRoR is training 15 ESRs in numerous aspects of clinical research from planning of research, to conduct and reporting, including PPI, via educational training from numerous international experts in the field. All 15 MiRoR ESRs are undertaking PhD research in their chosen area.

The 15 MiRoR ESRs have a range of different backgrounds from public health, pharmaceutical science, statistics, biomedicine, mathematics, to computer science, and linguistics. Their individual PhD research projects cover many different topics in research on research. Some examples are the development of advanced statistical methods, improving peer-review processes and scientific reporting, evaluating the impact of collective intelligence, methods for identifying research gaps and improving current methods to evaluate research quality.

Interactive training in research methods is a key concept of MiRoR; it is achieved via webinars, online journal clubs, and writing exercises. The biannual training event in particular is a core component of the MiRoR training programme. These events allow the 15 MiRoR ESRs to receive specialised training from experts in the field in topics ranging from science communication to computer programming. Their purpose is to equip the ESRs with a variety of tools and skills they can draw upon within their current PhD projects, and throughout their research careers. Despite the differences in the ESRs’ backgrounds and individual PhD projects the MiRoR training programme, enables the 15 ESRs to gain a wide range of methodology knowledge and skills relevant to their individual research projects and across the various biomedical scientific disciplines.

In March 2017, the University of Liverpool hosted a MiRoR training event, with sessions dedicated to PPI in research and communication of research to the wider public. Multidisciplinary teams, which included patient and public representatives, delivered both sessions, educating the MiRoR ESRs, consortium members in attendance and students and researchers invited from the University of Liverpool. Applied training included the importance of PPI contributors to clinical trial research from design to conduct and dissemination of findings. Applied workshops dedicated to qualitative research skills including interviewing and focus groups, which can be used to enable patient participation in research were also provided.

At the latest MiRoR meeting held at the University of Split in Croatia (2nd -3rd October 2018) a patient and a funder were invited to introduce the ESRs and their wider networks to the merits and importance of including patients and the public in every aspect of their research. Over the course of 2 days, both speakers delivered presentations to a general audience comprising members of the MiRoR consortium and students and professors from the University of Split Medical School. They also engaged in round table discussion with the 15 MiRoR ESRs along with a number of professors from the MiRoR consortium. This provided a unique opportunity to further discuss and reflect on the advantages and challenges of PPI in methodological research, building on the presentations.

In the following, we first summarise the main messages from the talks and the round table interactions and then discuss the ESRs’ views on PPI in research.

## What is waste in research? A patient perspective

During his presentation and subsequent discussions Mr. Richard Stephens, Chair of the National Cancer Research Institution’s (NCRI, a partnership of UK cancer research funders) Consumer Forum, brought a patient perspective on how to avoid waste in research. He described how wasted research is research that has the potential to be of value to patients’ lives but due to inadequate design, reporting, dissemination or lack of further research, it never translates into healthcare improvements. In addition, waste in research also relates to resources such as “*unused data and samples, and irrelevant research questions and findings*”. Loss of resources can occur at numerous junctures. Other issues described in the literature include, inappropriate design and methods, biased research and direct losses such as time and money [1, 5, 6], all of which have potentially negative implications for the end users of the research evidence.

### Actions and initiatives

As the advantages of PPI have gained recognition so has the role of patient organisations and communities. Mr. Stephens is a patient, he is also chair of one such organisation and, he is co-Editor-in Chief of an academic journal (BMC Research Involvement and Engagement). Thus, he is in a well-placed and unique situation to talk to and educate the MiRoR network about PPI. With Mr. Stephens, the interactions centred on two key areas; his own personal experience as a patient and what that meant to him and the wider initiatives that he has been involved in from academic journals to collaboration with industry.

He talked about how to include patients in the peer review process drawing on his experience as co-Editor-in-Chief. As a number of the MiRoR projects are exploring peer-review processes and journal editing this topic proved engaging and relevant. Discussions centred on the different facets to consider in the process. From recruitment of reviewers, to training needs and provision, to difference in topics of interest between patient and academic peer reviewers. Concerns were raised that patients might not be willing to review items that did not align with their priorities, beliefs and values and conversation ensued about whether the peer review process should be a collaborative process between patients and researchers. This would allow all perspectives to be discussed collectively.

He also introduced the group to the NCRI Consumer Forum, and highlighted the work and collaborations (with academia, industry and government bodies) that they have undertaken to facilitate better research. They view themselves as “*patients and public who are volunteers delivering professional standards in a professional environment*”. They view their role as the opportunity to “add value” to the research environment by “helping produce *good research*”, which is research that benefits patients. To achieve these objectives they provide training of patients to enable their full inclusion in research studies from being committee members to writing academic articles. This enabled discussion regarding whom to involve in PPI, what level of training and experience is needed and how their role differs from that of academic researchers. There was conversation about what are the most appropriate studies and time points in which PPI should be utilised, with multiple different viewpoints expressed regarding the different methods of involvement and the many roles a patient can have.

### Prioritisation of hot topics

Mr. Stephens talked about the differences in research priorities, and the importance of the outcomes measured, between patients and researchers. These differences can lead to irrelevant and redundant research studies and evidence, leading to waste in research, if the outcomes that truly matter to patients are omitted. For the members of the NCRI Consumer Forum “hot topics for research are early diagnosis, follow-up and quality of life”, whereas researchers in academia are often focussed on outcomes such as survival. Cancer research has made numerous diagnostic and therapeutic advancements, which have led to increased survival for several cancers and many cancer patients now live with comorbidities [29–31]. The prioritisation of research and measurement of outcomes surrounding patient experience and quality of life are becoming ever more important. Thus, there is a need for collaboration at that early stage of research. The NCRI is looking to address this through their initiative: “Living With and Beyond Cancer”, in collaboration with the James Lind Alliance. Together, they aim to identify and rank the “top ten relevant research priorities” from cancer patients and health care professionals and to work with funders to implement them in future research.

## Improving research integrity: a funder’s perspective

Dr. Matthew Westmore, was from the National Institute for Health Research (NIHR). He brought a funders’ perspective on waste in research, research integrity and the role of patients and the public in delivering better clinical research. Dr. Westmore encouraged consideration of how we can achieve the highest standards of rigour and integrity in all aspects of research. This includes the reduction of waste in research by incorporating PPI. By drawing on the “Concordat to Support Research Integrity” [32] during his presentation, Dr. Westmore described the importance “efficient research regulation and delivery” and the “effective dissemination of findings”. He illustrated how research integrity can be achieved by ensuring: “i) relevance and expressed need to setting justifiable research priorities, ii) excellence in research design, analysis and management, and iii) openness to ensuring methods and findings are accessible, complete and usable”. Later during the round table discussions the ESRs were able to follow up on this and further discuss the various stages involved in research applications and where PPI fits in.

### Actions and initiatives

He introduced the group to the various work the NIHR undertakes to reduce waste in research via the inclusion of PPI and how this helps determine the allocation of funds for research proposals. Every year the NIHR receives thousands of funding applications, however, it is not always a straightforward task to prioritise the research projects and decide which ones to fund. Thus, the NIHR work with a multidisciplinary panel of experts including the public, to consider applications. The feedback received leads to the creation of a short-list for further evaluation. In this process, one key factor for consideration is the impact of the research, i.e. what will the impact for patients be and how to maximise it. It is the hope of the NIHR that “every patient in the country has the opportunity to get involved in research”. The NIHR “Research Design Service” offers advice and support to applicants during the proposal writing process, which includes how to incorporate PPI into projects [33]. While not a part of Dr. Westmore’s presentation it is worth noting that the NIHR also has initiatives and strategies to support PPI contributors in becoming involved in research studies and raising research awareness, including the 2018 “I am Research” campaign.

### Mind the gap

NIHR have their own “research on research” team who works to identify what Dr. Westmore termed the “black hole of research gaps”. These gaps of unmet clinical needs are problematic, and can lead to the rise of non-evidence based treatments and interventions. He illustrated his point during a lively discussion on the use of crowdfunding to raise funds for “quack” cancer remedies such as “coffee enemas” [34]. Furthermore, the NIHR engage with the work of other bodies when considering how to prioritise needed research. This includes the JLA and the National Institute for Health and Clinical Excellence (NICE), both of whom collaborate with patients and members of the public. The JLA, as mentioned above, develops priority questions for research recommendations. NICE develops evidence based clinical and social guidelines; they also make “research recommendations” for areas in which they found insufficient evidence in the guideline development process. NIHR considers the recommendations of both these organisations when awarding funding.

## Journal Club

Within the diverse group of MiRoR fellows, there are varying levels of understanding and exposure to PPI in clinical research. There is an overall acknowledgment of the value and importance of PPI in research in MiRoR. However, unlike primary clinical research projects where patients are recruited as participants, our projects cover methodological issues in different phases of research from planning, to conduct, reporting, and peer-review. Hence, variation in implementation and applicability of PPI exists across the programme, thus we took an opportunity to reflect on PPI in our research in a recent journal club.

At the virtual journal club hosted in late October 2018 amongst the 15 MiRoR ESRs and a MiRoR network professor (PRW), we drew on our training events as outlined above and discussed a) whether it would have been possible to do things differently in our previous work and b) how we can implement PPI in ongoing and future work. Below we outline some key examples of PPI in individual MiRoR projects, thus illustrating the importance of educating ESRs in PPI and its merits, including PPI in methodological research.

### PPI in MiRoR; the potential and the limitations

One researcher (ESR 3) is exploring methods for including participants (patients and health professionals) in core outcome set (COS) development. A COS is an agreed minimum set of outcomes or outcome measures, it is a recommendation of what should be measured and reported in all trials in a specific area. A patient research partner has been involved in the qualitative study, in which patients will be interviewed, from the design and conduct, through to the analysis of findings. The next steps within this project include the co-production of a plain language educational tool, by a panel of researchers and patients. This project is the most prominent example of where there is a role for meaningful PPI in its conduct, as the research directly investigates the perspectives of patients and members of the public and explores methodological aspects in an attempt to improve their experiences and participation in COS development.

However, in light of the training provided by MiRoR in Liverpool and Split, some of the other ESRs reflected on whether PPI earlier in their projects would have also been useful. For example, ESR 1’s project is exploring methods for identifying and displaying research gaps. PPI in the planning phase to define the terminology used for shaping the project development may have been helpful:

*“We consulted different experts in the field; looking back it would have been extremely useful to also ask patients and the public on what they thought about the term “research gap” to gather a comprehensive list on the different terms as understood by experts, patients and the public.”*

Another researcher (ESR 13) is investigating peer-review content and communication processes in biomedical journals. PPI and patient participation in data collection from patient peer-reviewers alongside the journal editors may have offered different input and insight:

*“Patient peer reviewers are also part of the peer review process, I should have collected data from them as well in order to have a more complete, multi-faceted and holistic representation of peer reviewers in biomedical journals.”*

In other projects, the future prospects of PPI have become more apparent. ESR 15’s project is investigating how to measure peer-review report quality in biomedical research and the ESR conducting the project will now include patient editors as participants in their survey:

*“I strongly believe that their point of view is fundamental in order to ensure that a biomedical study is relevant and important for the end users.”*

ESR 14 is performing a study aiming to improve authors’ adherence to reporting guidelines during the peer review process of a biomedical journal; to date the project has had no PPI. However, the student is currently thinking about methods in which they could present the findings of this study to relevant patients thus, enabling a collaborative discussion piece on the patient impact of the findings. In addition, ESR 2 is developing preferred study designs/strategies for evaluating biomarkers as medical tests. To date the project has focused on documenting what study designs have been used in previously conducted studies. “*As this was a literature study, I don’t think there was a meaningful scope for PPI, as I find it unlikely that the study’s impact on the public/patients and vice versa, would have been different by including PPI. Mainly due to the descriptive nature of this study*”. *However, I think that other stages of research in medical test evaluation should consider including PPI.* E.g.*, for defining a “decision” (risk/benefit) threshold for when a patient would be willing to receive a diagnostic or screening test. Because, in contrast to our initial literature study, this will most likely have implications for the studies that directly evaluate a test/biomarker”.*

For some projects however, the purpose of PPI in the design and conduct of the research is not evident, particularly those with a strong statistical focus, for example the projects of ESR 4 and ESR 6. The former, ESR 4 aims to develop software for trialists so that they can use statistical models to predict patient recruitment in clinical trials more accurately. The latter concerns causal claims in observational studies and is modelling the outcomes of kidney transplantation, as a case study. These outcomes were chosen from a COS that was developed in the area together with patients [35]. However, after the training and discussion during the journal club the ESRs do acknowledge that various research projects have included PPI contributors in quantitative projects such as Hannigan et al. [36] and that with planning, training and collaboration of both researchers and PPI contributors there is potential for successful collaborations.

Difficulties in incorporating appropriate PPI also exist in the projects with a strong computer science focus. ESR 11 is developing automated methods for detecting some types of distorted reporting / spin (i.e. presentation of research results as being more positive/significant than they really are) in scientific articles. The target population for that specific intervention is academic researchers, thus PPI is deemed unfeasible for this particular project. However, as a result of the interactions in Split, the ESR is eager to learn more about it in their field: “*It is interesting to think about PPI in medical and clinical natural language processing (NLP): what is done and how, what could be done to improve the relevance of research.”*

In summary, these examples show how several projects have discovered the usefulness and potential of PPI, for previous, ongoing or future work, through our training and exercises. ESRs appeared to confuse the juncture between involvement and participation, when thinking about PPI in their own projects. For those who had not considered PPI in their projects prior to this session they talked mainly in terms of how they could ask patients to participate in their research, rather in terms of involvement. This in particular points to the need for ESRs and other researchers to be aware that PPI can and should start much earlier in the research process, and is not solely reliant on whether the research will include direct patient participation.

Despite varying levels of PPI in the projects, a common thread to all the projects is that the outputs should lead to better allocation of resources and more immediate results, ultimately, benefitting the patients. Therefore, there is potential to collaborate with patient research partners in disseminating the impact of all projects to the wider public, via plain language blog posts, videos and other appropriate means.

### Closing the gap; challenges and concerns

Through the training and reflections, the group also identified some challenges and concerns regarding their individual projects. For example, ESR 5 is exploring the impact of mobilising collective intelligence in clinical research planning. They hope to include patients and members of the public in their work, however in a topic that is currently quite new and complex for the researchers involved they struggle to find the appropriate language and explanation to do so: “*I plan to involve patients in research planning; however as researchers we struggle to translate the complex process of research planning into understandable language that other researchers and patients and public can contribute to. It is challenging to define the task, and then find a way to describe and visualise the problem to help contributors understand what they have to do.”* ESR 2 also noted, *“I think that my current knowledge and experience in PPI would not be appropriate for meaningful implementation. Ideally, PPI should have been introduced earlier in my education.”*

The group also reflected on general challenges, concerns, and areas in which we feel more guidance and instruction is required for ESRs to understand and implement PPI. Some of these issues are also well documented in the literature. Concerns centre on the emergence of professional patients, patients who through training and continuous engagement achieve specialised, specific knowledge and profiles, and therefore may no longer be representative of the typical patients [17]. However, while it was acknowledged that it is not possible to ask PPI contributors to represent all patients; concerns about being as inclusive as possible remained, in particular considering ways of reflecting the diversity of the patient community and including under-represented patient groups [37]. Others mentioned tokenism and using PPI as a tick-box to satisfy their funder or institution [38]. Thus, the group believes there is a need to discuss and further investigate the appropriate balance between training and informing patients, while allowing them to retain and provide their unique perspectives and lived experience. This also links to the need for understanding regarding the various roles in PPI and the skillsets needed at various points of the research process [25].

The group also discussed the need for guidance and support strategies for researchers from relevant organisations such patient representative groups and funders. The ESRs discussed the importance of receiving support and guidance specifically on lay language and communication, recruitment and involvement of patients in research and peer review. This discussion also provided an opportunity to highlight some of the literature and guidance that currently exists [11, 39].

## Discussion

As a research network, we have a keen interest in furthering our understanding of PPI and the benefits it can bring. Our individual projects focus on methodological work, this brings different challenges and merits to implementing PPI compared to the usual junctures of clinical research. However, as training opportunities like the ones outlined above show, there are different steps we can take now and in the future to incorporate PPI at various stages of methodological work. We are also more considerate of the impact on patients our research will have and the importance of communicating our findings. To date our dissemination activities have predominantly focussed on the scientific community; we have active output in blogs, scientific journals, Twitter and newsletters. However, we can take steps to increase our collaboration with patient representatives and engagement with the wider public.

At the next training event (Barcelona, March 2019) the ESRs will receive training on how to communicate with patients and the public in plain language, thus, addressing some of the concerns described above.

Future training should also stress the importance of researchers “actively listening” to PPI contributors. Understanding the motivations and logic behind a contributor’s comments will enable researchers to ensure a more meaningful collaboration. As a number of ESRs pointed to uncertainty about how to implement PPI in research future learning opportunities could look specifically at ways of implementation such as co-production, we note such guidance already exists for PPI in primary research [40]. More investigation is also needed on training PPI contributors receive in methodological research to improve the current practice [41].

In further efforts to communicate MiRoR’s research with patients and members of the public, we are currently developing videos and flyers about each of the ESR projects, explaining the importance of the research and findings, and the potential impact that it will have on patients. Further examples of this include ESR 3’s qualitative interview study with patient participants. ESR3 will co-produce a plain language summary of the results for the use of patients and the public with the People and Patient Participation, Involvement and Engagement (PoPPIE).. Further, PoPPIE will be able to assist in disseminating this summary to appropriate patient organisations and groups.

The value and importance of education and support opportunities, such as the ones received to date are unparalleled, and likely to be beneficial to all ESRs in clinical and methodological research. We also believe initiatives specific to ESRs, such as consideration of supervision from a senior with PPI experience or that all ESRs and supervisors could consider whether having a patient research partner would be beneficial to the research.

However, while the focus is on ESRs in this commentary, we strongly believe that the benefits of involving PPI in methodological research applies to any stage of a researcher’s career.

## Conclusion

The rationale for the importance of PPI is indisputable, for both research value, quality and integrity. Yet, although there is a wealth of educational material and guidance on PPI, it can be challenging to conceptualize meaningful PPI for some aspects of methodological research, most often because the results are not as directly obvious to patients and the public as in primary clinical research. Nonetheless, education in PPI for ESRs such as the training provided by MiRoR is essential to increase understanding and enhance skills for its proper implementation and needs further prioritisation. To facilitate this opportunity for others ESRs outside of network such as MiRoR, we believe that PPI should be a fundamental educational topic in the academic graduate curriculum, supported by universities via courses and seminars on PPI alongside other scientific skills that are required of an early stage researcher.

## Data Availability

N/A.

## Abbreviations

COMET: Core Outcome Measures in Effectiveness Trials
COS: Core outcome set
ESR: Early Stage Researcher
JLA: James Lind Alliance
MiRoR: Methods in Research on Research
NCRI: National Cancer Research Institute
NICE: National Institute for Health and Care Excellence
NIHR: National Institute for Health Research
PPI: Patient and Public Involvement
